# Immersive Learning Design for Technology Education: A Soft Systems Methodology

**DOI:** 10.3389/fpsyg.2021.745295

**Published:** 2021-12-17

**Authors:** C. H. Wu, Y. M. Tang, Y. P. Tsang, K. Y. Chau

**Affiliations:** ^1^Department of Supply Chain and Information Management, The Hang Seng University of Hong Kong, Shatin, Hong Kong SAR, China; ^2^Department of Industrial and Systems Engineering, The Hong Kong Polytechnic University, Kowloon, Hong Kong SAR, China; ^3^Faculty of Business, City University of Macau, Macao, Macau SAR, China

**Keywords:** technology education, immersive learning, learning performance, radio frequency identification, soft systems methodology

## Abstract

Science, technology, engineering and mathematics (STEM) education is a globalized trend of equipping students to facilitate technological and scientific developments. Among STEM education, technology education (TE) plays a significant role in teaching applied knowledge and skills to create and add value to systems and products. In higher education, the learning effectiveness of the TE assisted by the immersive technologies is an active research area to enhance the teaching quality and learning performance. In this study, a taught subject of radio frequency identification (RFID) assisted by using mixed reality technologies in a higher education institution was examined, while the soft systems methodology (SSM) was incorporated to evaluate the changes in learning performance. Under the framework of SSM, stakeholders’ perceptions toward immersive learning and RFID education are structured. Thus, a rich picture for teaching activities is established for subject control, monitoring, and evaluation. Subsequently, the design of TE does not only satisfy the students’ needs but also requirements from teachers, industries, and market trends. Finally, it is found that SSM is an effective approach in designing courses regarding hands-on technologies, and the use of immersive technologies improves the learning performance for acquiring fundamental knowledge and application know-how.

## Introduction

Technology education (TE) aims to promote technological literacy to meet societal needs arising from technological developments such as radio frequency identification (RFID), the internet of things (IoT), and cyber-physical systems (CPS) (Mammes et al., 2016). As TE includes a broad spectrum of technologies as educational topics, it relies on other STEM (which stands for science, technology, engineering and mathematics) disciplines to communicate a complete picture of technologies’ implementation and applications. In higher education, TE is essential because it equips students with professional knowledge to meet the needs of the coming digital era. Given the practical importance of technology and its increasing popularity in various industries, the demand for technical skills and technology implementation professionals continues to increase. Therefore a well-planned TE model is essential to train such professionals. Effective technological teaching and training are active research topics despite the steadily increasing popularity of technological applications in commercial practice and the demand for technological professionals. Nkhoma et al. (2017) presented a case method for enriching students’ learning experience and drawing considerable attention for teaching purposes. Knezek and Christensen (2016) extended the “will, skill, tool” (WST) model to be a novel pedagogy for technology-intensive professional development. Teachers’ wills and skills were incorporated into state-of-the-art technology tools. For example, Usmeldi et al. (2017) adopted a “define, design, develop and disseminate” (4D) model of science subjects to facilitate students’ critical thinking as an open-ended learning approach. Consequently, the foundation of adaptive learning is established to cultivate the enhancement and intelligence in the learning sequence, learning content, and assessment.

Although many innovative learning designs are proposed, most educational institutions cannot get rid of lectures in the course design due to their effectiveness in disseminating subject content to students. Admittedly, traditional lecturing is not a promising teaching approach to put students in a passive learning position, in which students’ attention is easily distracted (BajakMay et al., 2014; Akram et al., 2021). Consequently, immersive learning is recently advocated in higher education to enable learning activities in a simulated and artificial environment (Pagano, 2013). It revamps the teaching and learning practices pedagogically from content engagement to cognitive engagement. Currently, more and more research studies investigated the effectiveness of immersive learning and its influence on students’ performance (Makransky and Lilleholt, 2018; Tang et al., 2020). The immersive technologies can simulate complicated engineering and science concepts to increase specific knowledge and skills absorption and retention. Subsequently, learners engaged in simulated objects can be equipped with solid experience to specific domain knowledge through simulation, game-based learning, reality technologies, and 360-degree videos (Maas and Hughes, 2020; Shadiev et al., 2021).

Furthermore, the learning behavioral data can be effectively collected in the immersive environment to enhance the quality of teaching and learning. However, there is a lack of a systematic methodology to structure the immersive learning design pedagogically in the context of the TE research. Moreover, an all-rounded TE model is rarely discussed. Although several studies revealed the power of immersive technologies in teaching and learning activities, their application for the RFID education is limited. Therefore, the implementation of immersive technologies in engineering subjects should be further enriched. In addition to immersive technologies, a comprehensive strategy to manage teaching resources and tools for the group of stakeholders is needed to satisfy the stakeholders’ requirements and concerns.

This paper proposes an immersive learning-based TE model by considering soft systems methodology (SSM) to consolidate various stakeholders’ perceptions for improving teaching quality and students’ learning performance. A subject about teaching RFID with the aid of mixed reality (MR) technologies was investigated to assess the proposed methodology, where the deployment of RFID systems and different tag orientations were simulated in an artificial environment. Subsequently, SSM was used to evaluate and improve RFID education in higher education, first capturing the perceptions of students and teachers and then making systematic pedagogical changes. Among various emerging technologies, RFID has been implemented widely in numerous industries, including logistics, supply chain management, healthcare, and manufacturing (Rahman et al., 2017; Wang et al., 2018; Motroni et al., 2021). It is thus considered a key technology for global industry and commerce, which has drawn industrial practitioners’ and researchers’ attention (Ramanathan et al., 2014). Consequently, the RFID education, which is essential and fundamental for the research on IoT and CPS, is selected for the investigation in this study. On the other hand, SSM is an action research method that helps stakeholders understand different aspects of real-world problems tackled through learning. In educational research, SSM was utilized to the course timetabling problem for the faculty of management in universities (Mehregan et al., 2012). It has also been applied to modify a teaching module to understand students’ perceptions and satisfy their needs (Warwick, 2008). Also, SSM has contributed to educational policy reformation (Soemartono, 2014). Nevertheless, the adoption of SSM in the STEM learning design with the aid of immersive technologies is limited, without which the immersive TE is not properly structured. Considering the growing demand for TE in higher education, this study aims to systematically structure the perceptions of TE stakeholders to improve the learning experience and quality. Therefore, an RFID course taught in a university in Hong Kong is examined to revamp the course design pedagogically with the aid of mixed reality (MR) technologies using SSM. Subsequently, descriptive and inferential statistics are summarized to evaluate the learning performance of using immersive technologies in RFID education. Overall speaking, the proposed work in this study incorporates MR technology into engineering education. At the same time, the entire implementation roadmap and learning eco-system are structured by using the soft system methodology. An immersive learning pedagogy is thus formulated and examined to customize the learning experience to students, particularly theoretical and practical knowledge. The entire learning process can be adaptive to the environment with emerging immersive technologies.

The remainder of this paper is organized as follows. Section “Literature Review” reviews the literature concerning TE in RFID, immersive technologies, and SSM with applications. Subsequently, the field’s research gaps and questions are summarized. Section “Mixed Reality Learning Environment for the Technology Education” presents the MR learning environment for technology education. Section “Soft Systems Methodology for the Immersive Learning Pedagogical Model” describes the research methodology for immersive TE development. Finally, the results and implications of the proposed model are discussed in Section “Statistical Evaluations and Discussion.” Finally, Section “Conclusion” presents the conclusion.

## Literature Review

This section firstly states the motivation and values of the RFID education, which is selected for the investigation by immersive technologies and SSM. Also, immersive learning for technology education is reviewed. SSM and its applications are then introduced to establish the research methodology in this study. Finally, the research gaps and questions are summarized in this section.

### Technology Education in Radio Frequency Identification

Although RFID has been widely applied in the education industry as an educational technology (Kurniali and Mayliana, 2014; Motoyoshi et al., 2016), research conducted on TE in RFID is relatively limited. Only a few RFID teaching and learning models can be implemented and benchmarked for institutions and companies. According to a previous study (Buyurgan and Mendoza, 2008), most current RFID training is designed for industrial applications or organized by industrial practitioners rather than academic institutions. Moreover, Cheng and Prabhu (2013) proposed a training approach that integrated RFID with an organization’s enterprise information system. Although businesses in some industries provide training courses, these are mainly designed for specific commercial applications of limited scope. In general, TE lacks well-established RFID teaching and learning performance measurement standards, resulting in low learning effectiveness and decreasing students’ attention (Wieman, 2018; Li and Jiang, 2021). Tiernan (2010) found that, rather than lectures, engineering students are interested in acquiring hands-on experience with practical applications to apply emerging technologies after graduation. Other researchers have suggested that engineering courses in higher education should integrate different learning methods to transfer theoretical and practical knowledge thoroughly. Furthermore, applying practical teaching and learning methods improves students’ perceived learning quality. Other researchers have also addressed the relationship between educational methods and students’ learning performance. Thus, the teaching and learning methods for RFID technology are important for arousing students’ interest and improving their learning performance. The studies reviewed above reveal a gap between industry best practices and theoretical academic work in RFID education.

### Immersive Learning for the Technology Education

There are four typical categories of immersive learning technologies: simulation, game-based learning, reality technologies, and 360-degree videos (Maas and Hughes, 2020). Among the above, reality technologies, including virtual reality (VR), augmented reality (AR), and mixed reality (MR), are promising to let learners immerse themselves in the simulated environment and experience the interaction between physical and virtual objects (Brigham, 2017; Markowitz et al., 2018). VR creates an entirely virtual world while allowing users to experience a highly realistic environment, which is a kind of illusion with the effect of visual, auditory, interactive and other sensory-stimulating elements. Its primary characteristic is that users only interact in the virtual world without light sources or real-world interactions. AR is an enhanced technology that is originated from VR, which is to augment virtual objects to the real environment. Users are expected to perceive the digital content generated by the computing devices, which acts as a communication medium to interact with the physical world. Furthermore, MR is a combination of VR and AR as a whole, where the digital content enables the capability to evaluate the surrounding environment in a 3D manner. Subsequently, digital objects generated by MR technologies can be realized in the physical world. Recently, it has been found that there is a huge potential to explore the value of reality technologies in education, for example, fashion design and physical education (Ding et al., 2020; Elfeky and Elbyaly, 2021). It shows that AR technologies can effectively assist the teaching process on complicated theories and concepts through object visualization. However, the AR technologies cannot analyze the 3D environment for realizing digital objects, and thus the MR technologies should be further exploited for technology education.

### Soft Systems Methodology and Its Applications

In contemporary educational research, several studies unlocked the power of data analytics and mining approaches, for example, cluster analysis and decision tree, to discover the hidden values of the educational data during teaching and learning activities (Al Mazidi and Abusham, 2018; Križanić, 2020). Further, the growth of social media and e-learning platforms creates more opportunities in the data collection related to teaching and learning activities. Thus intelligent pedagogies for engineering education can be established (Gañán et al., 2015; Su and Lai, 2021; Su and Wu, 2021). Consequently, engineering education is now transformed to incorporate multiple resources and tools to enhance learning quality. Apart from applying state-of-the-art analytics methods, a systematic approach to organizing teaching resources and methods for satisfying requirements of the stakeholders, such as students, teachers, and companies, is needed to enhance the practicality of new pedagogies. Soft systems methodology (SSM) is a systematic approach to organizational process modeling for problem-solving and the management of change (Wilson and Van Haperen, 2015; Hanafizadeh et al., 2021). SSM is capable of finding improvements from complex problems and situations (Checkland and Poulter, 2020). SSM was originally treated as a modeling tool in the business sector, but it was further developed as a learning and training development tool to explore “messy” situations (Holland and Garfield, 2016). It can capture diverse stakeholders’ perspectives which can be addressed in both “hard” and “soft” aspects of a problem. In short, it allows stakeholders to investigate better, describe and understand the complexity of human activities and interactions on specific problems. Generally speaking, there are seven independent stages in the SSM to formulate an iterative process (Checkland and Poulter, 2020). In stage 1, the scope of the problem and key players interested in the organization’s function and objectives are identified. In stage 2, problems are illustrated by a “rich picture” to present the structures, processes, relationships and issues relevant to the problematic situation. In stage 3, the nature of the chosen system is articulated, including a rigorous description of the “what,” “when,” and “who” of the chosen system. Typically, the chosen system is described by six major elements, namely (i) customers, (ii) actors, (iii) transformation process, (iv) Weltanschauung (German for “comprehensive worldview”), (v) owner, and (vi) environmental constraints, which are generally abbreviated as “CATWOE.” Table 1 shows the customized CATWOE for the educational research to assist the pedagogical development. In stage 4, a conceptual model of the defined system is developed, while the comparison and debate of the conceptual model among stakeholders are conducted in stage 5. Desirable, feasible and possible changes are outlined in stage 6. Finally, the changes are implemented in stage 7.

**TABLE 1:** Elements of CATWOE for engineering education.

Element of CATWOE	Customization for the engineering education
Customers	Students
Actors	Teachers/Lecturers
Transformation	Teaching and learning process
Weltanschauung/Worldview	Incorporation of the emerging technology, for example, immersive technologies, in the teaching and learning activities of the engineering education
Owners	Top management in the institutions
Environmental Constraints	Market requirements of technical professionals, evolution of the technological know-how

In the field of education research, the values of the SSM can be further extended to refine the courses and learning design pedagogically in academic institutions. Hindle (2011) applied SSM to present a module blueprint to both students with pre- and post-experience in case scenarios, which demonstrates that SSM is an all-purpose approach to outline the module. Specifically, with the aid of the SSM, the design and evolution of teaching modules can be facilitated to address both “hard” and “soft” aspects of the learning experience. SSM can capture stakeholders’ perspectives while addressing the “hard” and “soft” aspects of the learning experience can facilitate the design and evolution of teaching modules in academic institutions. Yadin (2013) applied SSM to design an information systems course, while it was proved that students’ perception and understanding were successfully enhanced. Most existing studies focus on the technological deployment for the typical immersive technology-based teaching, regardless of the management of change. From traditional teaching activities to immersive technology-based teaching, the effective transition of the teaching resources and tools should be considered to enhance the teaching quality and students’ learning performance. Therefore, the SSM is the theoretical ground to facilitate the effective transition to the immersive learning environment. Therefore, it is believed that SSM can contribute to pedagogy in technology education, particularly in MR-based RFID education.

### Research Gap and Questions

Due to the rapid growth of state-of-the-art technologies, RFID technology is regarded as one of the key technologies to be implemented in various industries, such as logistics and supply chain management, to enhance the effectiveness and efficiency of their daily operations. Engineering education for RFID technology is essential to satisfy the increasing need for RFID implementation and maintenance. At the same time, most industrial practitioners and institutions are eager to design and develop an all-rounded course for training RFID experts. The power of immersive technologies can be fully revealed to develop effective teaching and learning methods for RFID technology in higher education. Thus learners can effectively immerse in the artificial environment for the RFID deployment. Beyond deploying immersive technologies in teaching and learning activities, unstructured and hidden problems from stakeholders’ perspectives should be considered to manage teaching resources and tools effectively. To fill this gap, the SSM is customized for the educational research in this study. Under the SSM framework, the effectiveness of using immersive technologies in teaching theoretical and practical knowledge should be evaluated. Overall, two research questions (RQ) in this study are summarized as follows:

***RQ1*** *: In terms of fundamental technological knowledge, what effect does the learning performance have on the MR-based teaching and learning method?*

***RQ2*** *: In terms of practical knowledge of technical applications, what effect does the learning performance have on the MR-based teaching and learning method?*

## Mixed Reality Learning Environment for the Technology Education

In this section, the MR learning environment for technology education is illustrated such that students can be immersed in the simulated environment for experiencing the system deployment and application.

### Architecture for Mixed Reality-Based Education

**FIGURE 1:**
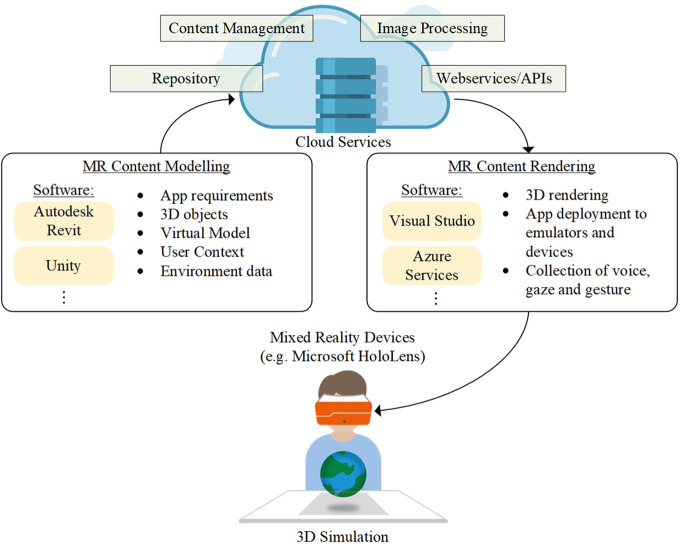
Architecture of mixed reality (MR)-based Technology Education.

Mixed reality applications are designed and developed to establish the MR-based learning environment, where the generic architecture of MR-based education is shown in Figure 1.

For deploying the designated applications on MR devices (e.g., Microsoft HoloLens), MR content modeling and rendering are two essential parts connecting to existing cloud services. In the MR content modeling, some 3D modeling engines, such as AutoDesk REVIT and Unity, can be adopted to build 3D objects according to application requirements. Also, user context and environment data are included to simulate virtual objects and content in reality effectively. For example, through the use of cloud services, such as content management, repository, image processing, and web services, the pre-requisites and toolkits of the MR applications are effectively managed to build the applications for 3D rendering. For rendering the 3D content, web and app development tools, such as Visual Studio, can compile the software package to MR devices and emulators for implementation. For placing holograms in the surrounding environment, the elements of lighting, landmarks, user movement, and Internet connection should be stabilized to maximize the hologram quality. Finally, based on the above architecture, the MR applications for technology education can be constructed to simulate 3D objects, such as hardware installation and system deployment.

### Immersive Environment for the Radio Frequency Identification Education

**FIGURE 2:**
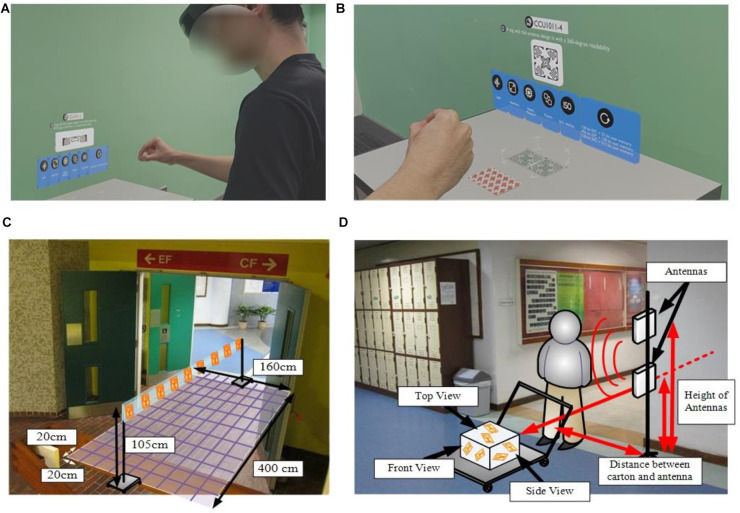
Deployment of mixed reality in radio frequency identification (RFID) education; **(A)** wearing HoloLens for RFID education; **(B)** displaying tag and antenna information in a virtual environment; **(C)** interrogation zone analysis (IZA); **(D)** tag placement analysis (TPA).

In the RFID education, fundamental knowledge on RFID is discussed and disseminated at the beginning, where several RFID applications are illustrated to stand out the value of the technology. MR technologies can visualize the fundamental knowledge of RFID, such as various types of tags and antennas (Figures 2A,B). To achieve the MR implementation in RFID education, the MR headset, HoloLens, is adopted in this study to visualize the tag and antenna information in the virtual environment. By wearing the headset, the students can read the details, such as tag type, dimension, and memory size, for the particular tags, where the HoloLens can identify the tags based on the tag shapes. By using MR technologies, students can effectively understand the details of various tags and antenna shapes. Apart from the theories and abstract knowledge, interrogation zone analysis (IZA) and tag placement analysis (TPA) are two essential components for the deployment of RFID (Jankowski-Mihułowicz and Wêglarski, 2017; Hashmi and Sharma, 2020). For the former analysis, students must set up the RFID tags and gateway to examine the tag response rates and signal coverage region based on setting different antennas, as shown in Figure 2C. For the latter analysis, the tag orientation and placement are critical to the RFID system performance. Thus, the optimal location and orientation to place tags should be obtained, as shown in Figure 2D.

Compared with the traditional laboratories for the above experiments in a limited time, a team of assistants is required to set up the hardware to achieve the analyses so that students cannot comprehensively experience the RFID deployment and installation in a real-life environment. With the aid of MR, the 3D illustration of the experimental setup and procedures can be visualized to deepen students’ understanding of the analyses. In addition, students can handle the experiments mainly by themselves to effectively understand the motivations and process of the analyses. Although the use of MR is promising in technology education, the differences in learning performance before and after the use of MR technologies should be investigated, while the pedagogical model for the RFID education should be formulated.

## Soft Systems Methodology for the Immersive Learning Pedagogical Model

**FIGURE 3:**
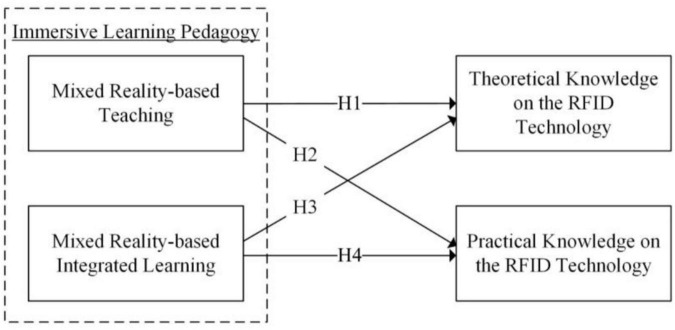
Hypothetical model of the immersive learning pedagogy.

This study applied SSM as the core methodology for reviewing and improving the existing pedagogical model for RFID education. To demonstrate the application of SSM, the RFID course with the use of MR technology in the Faculty of Engineering of the Hong Kong Polytechnic University in Hong Kong was selected as the case study. In addition, a hypothetical model is proposed to evaluate the learning performance of immersive learning, as shown in Figure 3. In the immersive learning pedagogical model, MR-based teaching and MR-based integrated learning are evaluated by measuring their theoretical and practical knowledge acquired after the teaching and learning activities.

### Step 1: Data Collection

To develop a rich picture that gathers information about a complex situation (Berg and Pooley, 2013), quantitative and qualitative data from students, lecturers and the university were collected. The data include a teaching plan and syllabus, which describe the requirements of the course design, internal discussions between lecturers, and assessment criteria to evaluate the students’ learning effectiveness with/without using MR technologies in learning. Thirty-five students who took the RFID course in Semester 2 of the academic year 2018/2019 and did not have prior RFID knowledge were selected as subjects in this study. The students were randomly assigned to five mutually exclusive groups and received RFID education for 13 weeks using different learning methods, including lectures, tutorials, seminars, laboratories and revision exercises. In addition, each student was asked to complete two different RFID assessment questionnaires containing thirty multiple-choice questions before the first teaching session (baseline measurement) and after the last class (post-test measurement), respectively. The objective of the module was to equip students with fundamental RFID knowledge and practical RFID know-how for being RFID professionals. Therefore, half of the assessment questions are related to fundamental knowledge, while another half is related to practical know-how.

Regarding the collection of the data collection (Liu, 2019), content management tools for e-learning, such as Moodle and Backboard, are used to set up the prior and post-assessments for students. Beyond the class performance, various control variables, such as attendance, nationality, and gender, can be considered to support the data analysis to generate meaningful insights for education research. The effectiveness of the adoption of MR technology was evaluated by the change in students’ performance in the RFID assessment questionnaire after the 13 weeks of teaching. The baseline measurement results are summarized in Table 2, while the group-based baseline measurement is summarized in Table 3.

**TABLE 2:** Baseline measurements for learning performance.

	Fundamental RFID knowledge	Practical knowledge
N	35	35
Minimum	0	1
Maximum	6	5
Mean	2.91	2.80
Std Dev	1.442	1.023

*Remark: N refers to the number of samples; Std Dev refers to the standard deviation.*

**TABLE 3:** Group-based baseline measurements for learning performance.

	Fundamental RFID knowledge			Practical knowledge		
Learning mode	Mean	*N*	Std Dev	Mean	*N*	Std Dev
Lecture	3.57	7	1.718	3.14	7	0.690
Seminar	2.86	7	1.345	3.29	7	1.113
Revision Exercise	3.14	7	1.345	3.00	7	0.816
Tutorial	2.86	7	1.069	2.43	7	1.397
Laboratory	2.14	7	1.676	2.14	7	0.690
Total	2.91	35	1.442	2.80	35	1.023

The baseline measurement results (Table 2) show that the students generally had insufficient RFID-related knowledge during course enrolment, with an average score of 2.91 out of 15 for fundamental RFID knowledge and 2.80 out of 15 for practical knowledge. Table 3 reflects the 35 students’ random assignment to the five mutually exclusive groups. The students’ baseline performance was relatively consistent among the groups (Table 3). In this investigation, the most appropriate learning model for RFID education in higher education is determined by adopting MR technologies and SSM. The summary of post-test measurements for fundamental RFID knowledge and practical knowledge are summarized in Table 4, while the group-based measurements are summarized in Table 5. In brief, it is found that students receiving lectures, seminars and revision exercises with the aid of MR technology generally performed better after the course with mean scores of 8.4 and 5.91 for fundamental RFID knowledge and practical knowledge, respectively. In addition, the students improved to a greater extent after the 13 weeks of teaching, in which the measurements in fundamental RFID knowledge and practical knowledge were improved by 5.49 and 3.11, respectively. For learning the fundamental RFID knowledge, lecturing, seminars, and revision exercises were the most effective measures; for learning practical knowledge, tutorials and laboratories generated the most positive impact.

**TABLE 4:** Post-test measurements for learning performance.

	Fundamental RFID knowledge	Practical knowledge
N	35	35
Minimum	1	4
Maximum	13	10
Mean	8.40	5.91
Std Dev	3.031	1.541

*Remark: N refers to the number of samples; Std Dev refers to the standard deviation.*

**TABLE 5:** Group-based post-test measurements for learning performance.

	Fundamental RFID knowledge			Practical knowledge		
Learning mode	Mean	*N*	Std Dev	Mean	*N*	Std Dev
Lecture	10.71	7	1.254	4.86	7	0.690
Seminar	9.86	7	1.464	5.00	7	0.577
Revision Exercise	10.14	7	1.773	5.00	7	0.577
Tutorial	6.14	7	1.676	7.71	7	1.496
Laboratory	5.14	7	3.436	7.00	7	1.291
Total	8.40	35	3.031	5.91	35	1.541

In addition to the descriptive statistics, inferential statistical analysis was applied to test the following hypotheses:

***Hypothesis 1 (H1)*** *: The five MR-based teaching methods are equally effective for fundamental RFID knowledge.*

***Hypothesis 2 (H2)*** *: The five MR-based teaching methods are equally effective for practical knowledge.*

For examining the above hypotheses, a non-parametric Kruskal–Wallis one-way ANOVA is used to test the established hypotheses, designed to compare the means of *k (k* > *3)* populations when the populations are not normally distributed with unequal variances. Thus, the learning performance is investigated using the above method at the 0.05 significance level in the SPSS environment. Table 6A summarizes the statistical analyses for fundamental RFID knowledge (H1) and practical knowledge (H2), respectively. Significant differences are observed between the students’ improvement (i.e., the effectiveness of the five MR-based learning methods) among the five teaching and learning methods, with *p*-Values < 0.001 for fundamental and practical knowledge. By using the ANOVA, *post hoc* comparisons (10 pairwise comparisons) were conducted. As shown in Tables 6B, C, we found significant differences between six pairwise comparisons (laboratory vs seminar, laboratory vs revision exercise, laboratory vs lecture, tutorial vs seminar, tutorial vs revision exercise and tutorial vs lecture) for both fundamental and practical knowledge, with *p*-Values < 0.05. The above results imply that the six pairs contribute to the differences observed in the students’ improvement (i.e., effectiveness of the five teaching and learning methods) among the five teaching and learning methods. Finally, as the five MR-based learning methods can contribute to effective fundamental and practical knowledge transfer, the most suitable MR-based learning method for RFID technology should integrate lecturing (i.e., lecture, seminar and revision exercise) and hands-on experience (i.e., laboratory and tutorial).

**TABLE 6A:** Kruskal–Wallis one-way ANOVA test for Hypothesis 1.

#	Null hypothesis	Test	Sig.	Decision
**H1**	Improvements of fundamental knowledge are the same across five MR-based teaching methods	Independent-Samples Kruskal-Wallis Test	0.000	Reject the null hypothesis
**H2**	Improvements of application know-how are the same across five MR-based teaching methods	Independent-Samples Kruskal-Wallis Test	0.000	Reject the null hypothesis

**TABLE 6B:** *Post hoc* pairwise comparisons for Hypothesis 1.

Sample 1-sample 2	Test statistic	Std. error	Std. test statistic	Sig.	Adj. Sig.
Laboratory-Tutorial	0.571	5.379	0.106	0.915	1.000
Laboratory-Seminar	17.429	5.379	3.240	0.001	0.012*
Laboratory-RevisionExercise	17.429	5.379	3.240	0.001	0.012*
Laboratory-Lecture	18.500	5.379	3.440	0.001	0.006*
Tutorial-Seminar	16.857	5.379	3.134	0.002	0.017*
Tutorial-RevisionExercise	16.857	5.379	3.134	0.002	0.017*
Tutorial-Lecture	17.929	5.379	3.333	0.001	0.009*
Seminar-RevisionExercise	0.000	5.379	0.000	1.000	1.000
Seminar-Lecture	1.071	5.379	0.199	0.842	1.000
RevisionExercise-Lecture	1.071	5.379	0.199	0.842	1.000

Remarks: Each row tests the null hypothesis that the Sample 1 and Sample 2 distributions are the same. Asymptotic significances (2-sided tests) are displayed. The significance level is 0.05, while the tests marked with * denote that the null hypotheses are rejected.

**TABLE 6C:** *Post hoc* pairwise comparisons for Hypothesis 2.

Sample 1-sample 2	Test statistic	Std. error	Std. test statistic	Sig.	Adj. Sig.
Lecture-Seminar	–0.500	5.329	–0.094	0.925	1.000
Lecture-RevisionExercise	–2.286	5.329	–0.429	0.668	1.000
Lecture-Laboratory	–17.357	5.329	–3.257	0.001	0.011*
Lecture-Tutorial	–19.500	5.329	–3.659	0.000	0.003*
Seminar-RevisionExercise	–1.786	5.329	–0.335	0.738	1.000
Seminar-Laboratory	–16.857	5.329	–3.163	0.002	0.016*
Seminar-Tutorial	–19.000	5.329	–3.565	0.000	0.004*
RevisionExercise-Laboratory	–15.071	5.329	–2.828	0.005	0.047*
RevisionExercise-Tutorial	–17.214	5.329	–3.230	0.001	0.012*
Laboratory-Tutorial	2.143	5.329	0.402	0.688	1.000

Remarks: Each row tests the null hypothesis that the Sample 1 and Sample 2 distributions are the same. Asymptotic significances (2-sided tests) are displayed. The significance level is 0.05, while the tests marked with * denote that the null hypotheses are rejected.

### Step 2: Formulation of a Rich Picture

**FIGURE 4:**
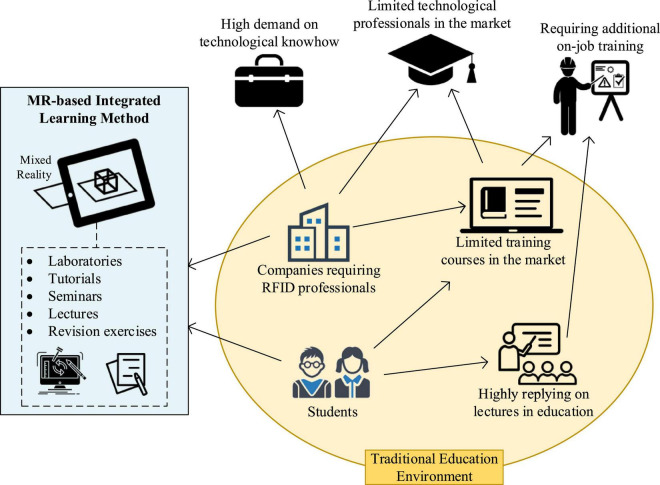
A rich picture of RFID education.

Based on the above statistical analysis, a rich picture can be constructed to describe the complex problem in designing an effective MR-based learning model, as shown in Figure 4. The rich picture highlights some emerging issues and questions that are particularly relevant to this research:

• Choices of RFID education and training available in academic institutions and the market for cultivating RFID professionals are limited. RFID knowledge is delivered through on-job training, and academic institutions organize only a few. So how can academic institutions, particularly higher education institutions in Hong Kong, prepare themselves to offer courses related to RFID technology?
• By their admission, some teachers are not aware of the effectiveness of teaching and learning method(s) for RFID technology, while lecturing is adopted as the main method. For example, how can teachers provide alternative teaching and learning methods for their students?
• Although existing research studies suggest integrating diverse teaching and learning methods for engineering education in higher education and emphasize the laboratory component as the most important aspect of RFID education. The most appropriate combination of teaching and learning methods has been under-researched. How can learning performance be improved effectively?

These issues and their solutions are interrelated. For instance, adequately addressing the third issue would provide teachers insights into designing a new syllabus to incorporate alternative teaching and learning methods (second issue). Furthermore, when the most suitable combination of teaching and learning methods can be identified, and its effectiveness examined (second issue), higher education institutions can benchmark teaching and learning methods and collaboratively establish standards for methods in RFID education (first issue). Finally, to illustrate the use of SSM in redesigning the case study RFID course, the third issue is focused on in this study.

### Step 3: Development of the Root Definition

To describe the nature of the problem on RFID education, the CATWOE (which stands for customers, actors, transformation, *Weltanschauung*, owners and environmental constraints) is adopted to identify the significant elements of the root definition shown in Tables 7A, B. The root definition describes the system of interests using the SSM terminologies. The elements of the CATWOE method are explained in response to the problems observed in RFID education.

**TABLE 7A:** The root definition of the radio frequency identification education model.

Elements of CATWOE	Element description(s)
Customers	The students enrolled in the RFID technology course
Actors	The course teacher(s) and students (in laboratory and tutorial exercises)
Transformation	Imparting fundamental and practical RFID knowledge using an integration of various suitable teaching and learning methods
Weltanschauung	Through integrated teaching and learning methods, students can effectively acquire both fundamental and practical knowledge With standardized RFID teaching and learning methods and environments provided by higher education institutions, graduates will be able to become RFID professionals to meet the increasing market demand for such professionals
Owners	Dean of Faculty of Engineering, Head of Department of Industrial and Systems Engineering, or Course Coordinator
Environmental constraints	University Grants Committee (which financially supports Hong Kong’s higher education institutions), university regulations, other courses enrolled by students, work, outside commitments and family

**TABLE 7B:** Revised teaching plan for the radio frequency identification education.

Teaching and learning method (in hours/session)	Schedule (in week #)	Student study effort (in hours)
Lecture (2 h)	1, 2, 4, 5, 7, 9	12
Tutorial (1 h)	1, 2, 4, 5, 7, 9	6
Laboratory (3 h)	3, 6, 8, 10	12
Seminar (2 h)	6, 11	4
Revision Exercise (2 h)	12	2
Assessment*	13	3
The total student study effort		39

*Not a change in the proposed teaching/learning method.

### Step 4: Construction of the Education Models

**FIGURE 5:**
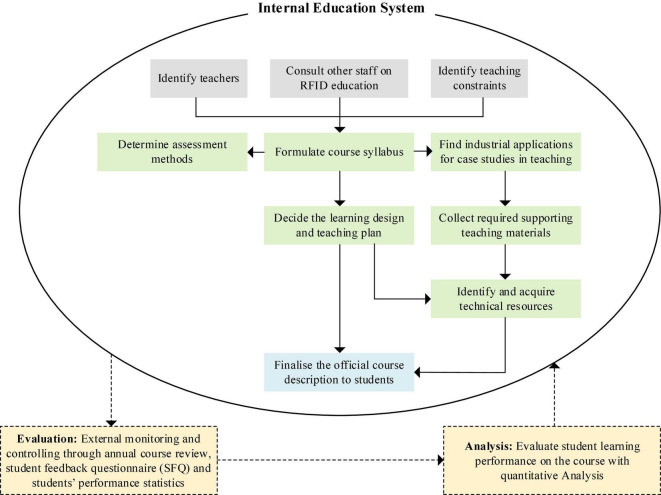
Graphical illustration for the learning design process.

After defining the root definition of the chosen system, we developed a conceptual model of RFID education to describe the activities that contribute to its transformation. Figure 5 shows the conceptual model of the TE learning design process, demonstrating the connection between the internal education system and external monitoring. The conceptual model reflects the activities and their sequences. The large ellipse represents the complex system. The various activities to be performed are connected by arrows that indicate their sequence of occurrence. Furthermore, SSM includes activities that monitor and control the system to take corrective actions when the expected results are not achieved. In addition, monitoring and control activities maintained the system effectively to be designed correctly to achieve its purpose in implementing transformation. Moreover, the activities described in the conceptual model can be explored more deeply when considered as sub-systems.

**FIGURE 6:**
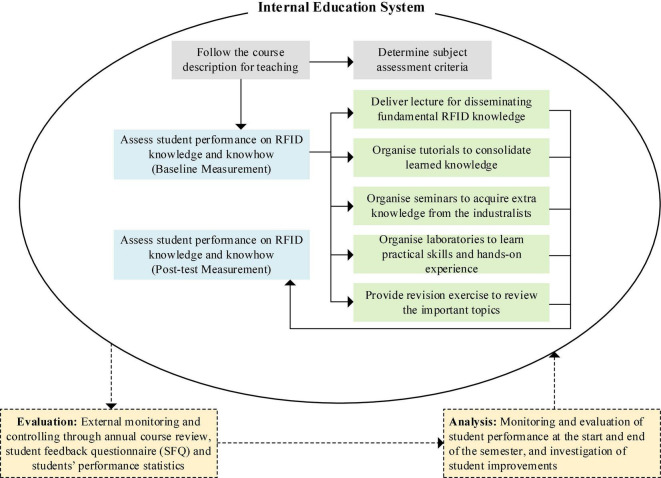
Graphical illustration of the RFID education model.

To adapt to the demand of RFID professionals in the market, an RFID education model derived from the basic TE model is developed, focusing on teaching and learning for RFID technology (Figure 6). Changes in teaching and learning methods to the course structure are introduced to provide fundamental and practical knowledge for students in higher education institutions. Effective MR-based learning methods, including lectures, seminars, revision exercises, tutorials and laboratories, should be integrated as a whole. Specifically, it is recommended that students first attend lectures to acquire fundamental knowledge and immediately participate in tutorials to consolidate the knowledge acquisition process. Also, students are suggested to participate in laboratory sessions to apply the fundamental knowledge they had learned, acquire additional practical skills, and obtain hands-on experience. In addition, guest speakers would be invited to deliver seminars to transfer real-life knowledge and case studies to students. Revision exercises were designed to provide students to evaluate their understanding of specific knowledge and theories. Finally, lectures, laboratories, and seminars were designed to be iterative to promote continual learning and consolidation in the subject syllabus. Students’ fundamental and practical RFID knowledge is assessed with different assessment tasks to regularly review learning performance and identify areas of improvement in RFID education.

### Step 5: Debate and Discussion With Stakeholders

The proposed RFID education model was compared with the existing practice and requirements, and debates among stakeholders were arranged to capture their perspectives concerning RFID education. It aims to revamp the course structure and content in a practical and commonly agreed manner. This stage also provides a chance for the stakeholders to express their ideas and comments on the education model in terms of teaching materials, assessment methods, and hands-on experience sufficiency. Consequently, feasible and desirable improvements on the RFID education based on stakeholders’ perspectives can be outlined.

### Step 6: Proposal of Changes in the Teaching Plan

The original teaching plan provides only a formal series of lectures followed by assessment, while sessions for transferring practical knowledge are not included. The development of the aforementioned education model entails reviewing and revising the course’s teaching and learning methods. The updated teaching plan integrates an MR-based learning experience that allows students to acquire fundamental and practical knowledge in a simulated and artificial environment. Opportunities to experience RFID system deployment and RFID analyses at the first-person vision are now given. The revamped course structure also provides opportunities for students to gain practical skills and accumulate hands-on experience related to RFID technology. Therefore, students can be equipped with specific theoretical and practical knowledge to adapt to the emerging technological trends on RFID, IoT, CPS, etc. Learning performance and teaching effectiveness are then evaluated by assessment questionnaires tentatively scheduled to be held at the end of the course. The assessments are designed to provide feedback on students’ performance and suggestions for further refinements to the course structure.

### Step 7: Implementation of the Revised Radio Frequency Identification Education

In the final stage of SSM, the changes identified in Step 6 are implemented for improving the quality of the RFID education. Therefore, the transformation of the RFID course is performed over the summer break in the academic year 2019/20 and implemented for the first time in Semester 1, having been formally approved by the subject coordinator and program leader of the university. Consequently, the revised teaching plan for RFID education, as depicted in Table 8, are proposed and implemented.

**TABLE 8:** Summary of comparative analysis of the learning performance.

		Theoretical knowledge	Practical knowledge
**Baseline measurement**			
MR-based Lectures	Mean	3.39	2.56
	N	18	18
	Std Dev	1.577	0.984
MR-based Integrated learning	Mean	3.11	2.39
	N	18	18
	Std Dev	1.278	1.037
Total	Mean	3.25	2.47
	N	36	16
	Std Dev	1.422	1.000
**Post-test measurement**			
MR-based Lectures	Mean	10.17	5.00
	N	18	18
	Std Dev	1.338	1.138
MR-based Integrated learning	Mean	10.11	7.56
	N	18	18
	Std Dev	1.605	1.381
Total	Mean	10.14	6.28
	N	36	36
	Std Dev	1.457	1.799
**Overall Improvement**			
MR-based Lectures	Mean	6.78	2.44
	N	18	18
	Std Dev	1.003	1.097
MR-based Integrated learning	Mean	7.00	5.17
	N	18	18
	Std Dev	0.767	0.707
Total	Mean	6.89	3.81
	N	36	36
	Std Dev	0.887	1.653

## Statistical Evaluations and Discussion

In the past, the RFID course was dominated by lectures without particular emphasis on practical knowledge or hands-on experience about RFID technology. With enabling MR technologies in the revised RFID education model, the comparative analysis of students’ learning performance can be investigated to validate the effectiveness of MR-based learning. Also, the contributions and theoretical insights from this study are described in this section.

### Comparative Analysis of Mixed Reality-Based Radio Frequency Identification Education

The course structure changes were implemented and evaluated by considering the differences of students’ learning performance. Thirty-six students with no prior RFID knowledge and education enrolled in the RFID course in Semester 1 of the academic year 2019/20. These students were randomly assigned to two mutually exclusive groups that received RFID teaching using two different teaching and learning methods, namely (i) MR-based lectures only and (ii) MR-based integrated learning, for 13 weeks. The primary topics, teachers and students’ study efforts in terms of contact hours per week were the same. For the MR-based integrated learning, students are expected to receive the RFID knowledge from a mix of lectures, tutorials, laboratories, seminars and revision exercises, as illustrated in Table 8. Similar to the data collection in Step 1 of the SSM stated in Section “Soft Systems Methodology for the Immersive Learning Pedagogical Model,” the same set of RFID assessment questionnaires were distributed to students before the first class of teaching (baseline measurement) and after the last class (post-test measurement). Again, the effectiveness of the two sets of teaching and learning methods was evaluated by the change in students’ performance in the RFID assessment questionnaire after the 13 weeks of teaching. As shown in Table 9, the results of the comparative analysis are presented, including the baseline measurement, post-test measurement, and overall improvement. It is found that the hybrid use of MR technologies and integrated learning approach provides a significant improvement on learning for both theoretical and practical knowledge. It implies that the MR technologies are helpful to visualize 3D objects for assisting the lecturing process and beneficial to enhance the quality of tutorials, seminars, and laboratories. The students are more effective than before to acquire comprehensive theoretical and practical knowledge on RFID.

**TABLE 9:** Non-parametric test for improvement difference evaluation.

#	Null Hypothesis	Test	Sig.	Decision
H3	Improvements in theoretical knowledge are the same across two learning methods	Independent-Samples Mann-Whitney U Test	0.673	Retain the null hypothesis
H4	Improvements in practical knowledge are the same across two learning methods	Independent-Samples Mann-Whitney U Test	0.000	Reject the null hypothesis

*Remarks: Asymptotic significances are displayed, and the significance level is 0.05.*

Moreover, inferential statistical analysis was applied to determine a statistical difference between the two independent groups of students in RFID learning performance. As the sample included only 36 subjects, a non-parametric test is thus selected. According to Richardson (2010), the Mann–Whitney *U* test is a non-parametric statistical test that compares two independent or unrelated samples. Thus, we conducted a Mann–Whitney *U* test at the 0.05 significance level in the SPSS environment. As shown in Table 9, it is found that no significant difference in the learning performance for theoretical knowledge is observed between the two groups, with a *p*-Value = 0.673 (>0.05). However, a significant difference in practical knowledge learning performance between the two groups is observed, with a *p*-Value < 0.01. Generally speaking, the group receiving MR-based integrated learning performed significantly better than another group. Particular for learning practical knowledge, the use of MR-based integrated learning (mean improvement of 5.17 points) is better than another group that receives MR-based lectures only (mean improvement of 2.44 points). These results demonstrate the positive effect of the changes to the course structure proposed by the MR-based RFID education model. Finally, it implies that the MR-based integrated learning approach can improve current RFID education and training in higher education institutions to cultivate RFID professionals for the market effectively.

### Contributions and Theoretical Insights in Technology Education

This study explored effective technology education (TE) methods using SSM and MR technologies for an RFID course. Existing TE frameworks and models focus on conducting surveys, experiments and statistical analysis for various learning and teaching theories (Buckley et al., 2019; Priemer et al., 2020). They propose tailor-made structures and frameworks to construct new education theories and models to improve TE. However, such approaches are ill-suited to generalize a systematic structure to identify, construct, evaluate and provide feedback on TE content. In contrast, this study proposes the integration of SSM and MR and applies this combined approach to an RFID course.

On the one hand, this study can solve the problems of training new RFID professionals by providing an effective method for developing course structures and syllabi. The study establishes that rich pictures and education models are beneficial for industries and academic institutions. On the other hand, the study represents a novel implementation of SSM for creating MR-based TE models and theories in RFID courses. With the integration of SSM, complex organizational situations and problems in TE can be structured to consider the roles of various stakeholders in an organized and systematic manner. Thus, TE’s “soft” problems, including human activities, managerial changes and organizational issues, can be solved effectively by incorporating critical systems thinking in the solutions. Such an approach promotes the structured and systematic evolution of TE. Since not all problems can be solved by “hard” engineering techniques such as operations research, particularly in education, SSM has advantages for addressing problems in complex and ever-changing organizational and managerial situations. Such approaches can improve the design of education models and frameworks to meet the challenges of the learning and teaching process. Also, the effectiveness of the proposed education models can be established by incorporating evaluations and statistical analysis. According to the statistical findings in this study, it is revealed that the use of MR technologies can positively enhance the students’ learning performance in both theoretical and practical knowledge related to the RFID technology. Therefore, it is effective for students to understand the complicated concepts of the RFID technology, such as antenna design and tag orientation, using the MR devices, where the learning activities can be conducted in an immersive environment.

Furthermore, MR-based integrated learning under the soft system methodology is relatively effective in delivering practical knowledge to the students, compared with MR-based lecturing. In other words, students can effectively acquire practical skills on the RFID technology utilizing the MR-based integrated learning approach. The method proposed in this study can be extended to other areas of education and TE to construct models and theories with structured frameworks and systematic analysis for effective STEM education. Finally, the entire development and eco-system of technology and design education in both academic institutions and industries can be beneficial to advocate STEM education.

## Conclusion

This study addresses the academic and practical importance of the systematic organization of technology education (TE) by combining soft systems methodology (SSM) and mixed reality (MR) with statistical analysis to derive a novel pedagogical learning design. The research findings offer teachers and higher education institutions a conceptual model for technology education for future benchmarking. More importantly, this study provides a systematic approach for higher education institutions to review their current pedagogical practices and address any hidden problems in a commonly agreed manner. The SSM-based study can improve the quality of TE for better cultivating technological professionals in the market. The methodology and results of this study can be further extended to redesign other courses related to STEM education. The study’s primary limitation reflects the nature of SSM, in which the findings are only relevant to the places (or cases) being investigated, namely technology education for the RFID technology. This study establishes the usefulness of the SSM methodology for redesigning technology courses to disseminate theoretical and practical knowledge to students effectively. In addition, the materials and technology to be taught should be visualized by the MR technology, for example, the antenna design and experiment setup. Otherwise, the values of using MR technology in the teaching activities cannot be fully revealed. For future work, the proposed TE model should be further applied by more lecturers and institutions related to STEM subjects, particularly for some visualizable concepts and knowledge. Last, by not least, the research of the MR-based teaching and learning environment can be further explored and investigated to establish a comprehensive eco-system for learning in this digital era.

## Data Availability Statement

The raw data supporting the conclusions of this article will be made available by the authors, without undue reservation.

## Author Contributions

CHW contributed to the first draft of the article, conception, design of the study, and collected and analyzed the data. YMT contributed to the first draft of the article and VR environment. YPT contributed to data processing, performed the statistical analysis, and carried out the experiment. KYC secured the partial research, funding, and data collection and helped to shape the research. All authors contributed to manuscript revision, read, and approved the final version.

## Conflict of Interest

The authors declare that the research was conducted in the absence of any commercial or financial relationships that could be construed as a potential conflict of interest.

## Publisher’s Note

All claims expressed in this article are solely those of the authors and do not necessarily represent those of their affiliated organizations, or those of the publisher, the editors and the reviewers. Any product that may be evaluated in this article, or claim that may be made by its manufacturer, is not guaranteed or endorsed by the publisher.

## References

[B1] Akram H. Yingxiu Y. Al-Adwan A. S. Alkhalifah A. (2021). Technology Integration in Higher Education During COVID-19: An Assessment of Online Teaching Competencies Through Technological Pedagogical Content Knowledge Model. *Front. Psychol.* 12:736522. 10.3389/fpsyg.2021.736522 34512488 PMC8426343

[B2] Al Mazidi A. Abusham E. (2018). Study of general education diploma students’ performance and prediction in Sultanate of Oman, based on data mining approaches. *Int. J. Eng. Bus. Manag.* 10:1847979018807020. 10.1177/1847979018807020

[B3] BajakMay A. KaiserJun J. GrimmJun D. O’GradyJun C. O’GradyJun C. CleryJun D. (2014). *Lectures aren’t just boring, they’re Ineffective, too, study finds.* Available online at: https://www.sciencemag.org/news/2014/05/lectures-arent-just-boring-theyre-ineffective-too-study-finds#disqus_thread (accessed July 11, 2020)

[B4] Berg T. Pooley R. (2013). Contemporary iconography for rich picture construction. *Syst. Res. Behav. Sci.* 30 31–42. 10.1002/SRES.2121

[B5] Brigham T. J. (2017). Reality check: basics of augmented, virtual, and mixed reality. *Med. Ref. Serv. Q.* 36 171–178. 10.1080/02763869.2017.1293987 28453428

[B6] Buckley J. Seery N. Power J. Phelan J. (2019). “The importance of supporting technological knowledge in post-primary education: A cohort study.”. *Res. Sci. Technol. Educ.* 37 36–53. 10.1080/02635143.2018.1463981

[B7] Buyurgan N. Mendoza A. (2008). Creating a Learning Environment for RFID Education. *Decis. Sci. J. Innov. Educ.* 6 257–263. 10.1111/j.1540-4609.2008.00172.x

[B8] Checkland P. Poulter J. (2020). *Soft systems methodology, in Systems approaches to making change: A practical guide.* London: Springer, 201–253.

[B9] Cheng C. Y. Prabhu V. (2013). An approach for research and training in enterprise information system with RFID technology. *J. Intell. Manuf.* 24 527–540. 10.1007/s10845-011-0595-4

[B10] Ding Y. Li Y. Cheng L. (2020). Application of Internet of Things and virtual reality technology in college physical education. *IEEE Access.* 8 96065–96074. 10.1109/ACCESS.2020.2992283

[B11] Elfeky A. I. M. Elbyaly M. Y. H. (2021). Developing skills of fashion design by augmented reality technology in higher education. *Interact. Learn. Environ.* 29 17–32. 10.1080/10494820.2018.1558259

[B12] Gañán D. Caballé S. Conesa J. Xhafa F. (2015). An application framework to systematically develop complex learning resources based on collaborative knowledge engineering. *Int. J. Appl. Math. Comput. Sci.* 25 361–375. 10.1515/amcs-2015-0028

[B13] Hanafizadeh P. Mehrabioun M. Mostasharirad A. (2021). The necessary and sufficient conditions for the solution of soft systems methodology. *Philosop. Manage.* 20 135–166. 10.1007/s40926-020-00149-7

[B14] Hashmi M. S. Sharma V. (2020). Design, analysis, and realisation of chipless RFID tag for orientation independent configurations. *J. Eng.* 2020 189–196. 10.1049/joe.2019.0920

[B15] Hindle G. A. (2011). Case article—teaching soft systems methodology and a blueprint for a module. *Informs J. Comput.* 12 31–40. 10.1287/ited.1110.0068ca 19642375

[B16] Holland L. Garfield J. (2016). Linking research and teaching: an applied soft systems methodology case study. *Int. J. Inf. Technol. Syst. Approach* 9 23–38. 10.4018/IJITSA.2016070102

[B17] Jankowski-Mihułowicz P. Wêglarski M. (2017). Definition, characteristics and determining parameters of antennas in terms of synthesising the interrogation zone in RFID systems. *IEEE J. Radio Freq. Identif.* 2017 65–119. 10.5772/intechopen.71378

[B18] Knezek G. Christensen R. (2016). Extending the will, skill, tool model of technology integration: Adding pedagogy as a new model construct. *J. Comput. High. Educ.* 28 307–325. 10.1007/s12528-016-9120-2

[B19] Križanić S. (2020). Educational data mining using cluster analysis and decision tree technique: A case study. *Int. J. Eng. Bus. Manag.* 12:1847979020908675. 10.1177/1847979020908675

[B20] Kurniali S. Mayliana (2014). “The development of a web-based attendance system with RFID for higher education institution in binus university,” in *EPJ Web of Conferences*, Vol. 68 (France: EDP Sciences), 38.

[B21] Li J. Jiang Y. (2021). The Research Trend of Big Data in Education and the Impact of Teacher Psychology on Educational Development During COVID-19: A Systematic Review and Future Perspective. *Front. Psychol.* 12:753388. 10.3389/fpsyg.2021.753388 34777150 PMC8578735

[B22] Liu C. K. (2019). A holistic approach to flipped classroom: A conceptual framework using e-platform. *Int. J. Eng. Bus. Manag.* 11:1847979019855205. 10.1177/1847979019855205

[B23] Maas M. J. Hughes J. M. (2020). Virtual, augmented and mixed reality in K–12 education: A review of the literature. *Technol. Pedagog. Educ.* 29 231–249. 10.1080/1475939X.2020.1737210

[B24] Makransky G. Lilleholt L. (2018). A structural equation modeling investigation of the emotional value of immersive virtual reality in education. *Educ. Technol. Res. Dev.* 66 1141–1164. 10.1007/s11423-018-9581-2

[B25] Mammes I. Fletcher S. Lang M. Münk D. (2016). “Technology education in Germany in *Technology Education Today*,” in *International Perspectives*, eds de Vries M. J. (Münster, NY: Waxmann), 11–38.

[B26] Markowitz D. M. Laha R. Perone B. P. Pea R. D. Bailenson J. N. (2018). Immersive virtual reality field trips facilitate learning about climate change. *Front. Psychol.* 9:2364. 10.3389/fpsyg.2018.02364 30555387 PMC6284182

[B27] Mehregan M. R. Hosseinzadeh M. Kazemi A. (2012). An application of soft system methodology. *Proc. Soc. Behav. Sci.* 41 426–433. 10.1016/j.sbspro.2012.04.051

[B28] Motoyoshi T. Tetsumura N. Masuta H. Koyanagi K. I. Oshima T. Kawakami H. (2016). Tangible gimmick for programming education using RFID systems. *IFAC PapersOnLine* 49 514–518. 10.1016/j.ifacol.2016.10.608

[B29] Motroni A. Buffi A. Nepa P. (2021). A survey on indoor vehicle localisation through RFID technology. *IEEE Access* 9 17921–17942. 10.1109/access.2021.3052316

[B30] Nkhoma M. Sriratanaviriyakul N. Quang H. L. (2017). Using case method to enrich students’ learning outcomes. *Active Learn. High. Educ.* 18 37–50. 10.1177/1469787417693501

[B31] Pagano K. O. (2013). *Immersive Learning*. Alexandria, VA: American Society for Training and Development.

[B32] Priemer B. Eilerts K. Filler A. Pinkwart N. Rösken-Winter B. Tiemann R. (2020). A framework to foster problem-solving in STEM and computing education. *Res. Sci. Technol. Educ.* 38 105–130. 10.1080/02635143.2019.1600490

[B33] Rahman F. Bhuiyan M. Z. A. Ahamed S. I. (2017). A privacy preserving framework for RFID based healthcare systems. *Future Gener. Comput. Syst.* 72 339–352. 10.1016/j.future.2016.06.001

[B34] Ramanathan R. Ramanathan U. Ko L. W. L. (2014). Adoption of RFID technologies in UK logistics: Moderating roles of size, barcode experience and government support. *Expert Syst. Appl.* 41 230–236. 10.1016/j.eswa.2013.07.024

[B35] Richardson A. (2010). Non-parametric statistics for non-statisticians: A step-by-step approach. *Int. Stat. Rev.* 78 451–452. 10.2307/27919868

[B36] Shadiev R. Yu J. Sintawati W. (2021). Learning Activities Supported by 360-Degree Video Technology Have Positive Impact on Language Learning, Intercultural Communicative Competence Development, and Knowledge Sharing. *Front. Psychol.* [Preprint]. 10.3389/fpsyg.2021.766924 PMC8663917 34899512

[B37] Soemartono T. (2014). Reconstruction of Education Policy in Jembrana Bali, Best Practices of Creative and Innovative Leadership using Soft Systems Methodology based Action Research. *Proc. Soc. Behav. Sci.* 115 269–282. 10.1016/j.sbspro.2014.02.435

[B38] Su Y. S. Lai C. F. (2021). Applying educational data mining to explore viewing behaviors and performance with flipped classrooms on the social media platform Facebook. *Front. Psychol.* 12:653018. 10.3389/fpsyg.2021.653018 33995212 PMC8116531

[B39] Su Y. S. Wu S. Y. (2021). Applying data mining techniques to explore user behaviors and watching video patterns in converged IT environments. *J. Ambient Intell. Humaniz. Comput.* 2021:6. 10.1007/s12652-020-02712-6 33425047 PMC7775737

[B40] Tang Y. M. Au K. M. Lau H. C. Ho G. T. Wu C. H. (2020). Evaluating the effectiveness of learning design with mixed reality (MR) in higher education. *Virtual Real.* 24 797–807. 10.1007/s10055-020-00427-9

[B41] Tiernan P. (2010). Enhancing the learning experience of undergraduate technology students with LabVIEW™ software. *Comput. Educ.* 55 1579–1588. 10.1016/j.compedu.2010.07.001

[B42] Usmeldi U. Amini R. Trisna S. (2017). The development of research-based learning model with science, environment, technology, and society approaches to improve critical thinking of students. *J. Pendidik. IPA Indones.* 6 318–325. 10.15294/jpii.v6i2.10680

[B43] Wang C. Chen X. N. Soliman A. H. A. Zhu Z. (2018). RFID Based Manufacturing Process of Cloud MES. *Future Internet* 10:104. 10.3390/fi10110104

[B44] Warwick J. (2008). A case study using soft systems methodology in the evolution of a mathematics module. *Math. Enthus.* 5 269–290.

[B45] Wieman C. (2018). “STEM Education: Active Learning or Traditional Lecturing,” in *Learning 4.0 : Advanced Simulation, Immersive Experiences and Artificial Intelligence, Flipped Classrooms, Mentoring and Coaching*, eds Salvetti F. Bertagni B. (Italy: Franco Angeli Edizioni), 10–15.

[B46] Wilson B. Van Haperen K. (2015). *Soft systems thinking, methodology and the management of change.* London: Macmillan International Higher Education.

[B47] Yadin A. (2013). Soft Systems Methodology in an Educational Context–Enhancing Students Perception and Understanding. *Int. J. e-Educat. e-Bus. e-Manage. e-Learn.* 3:351. 10.7763/IJEEEE.2013.V3.258

